# Structural Origins for the Loss of Catalytic Activities of Bifunctional Human LTA4H Revealed through Molecular Dynamics Simulations

**DOI:** 10.1371/journal.pone.0041063

**Published:** 2012-07-25

**Authors:** Sundarapandian Thangapandian, Shalini John, Prettina Lazar, Sun Choi, Keun Woo Lee

**Affiliations:** 1 Division of Applied Life Science (BK21 Program), Systems and Synthetic Agrobiotech Center, Plant Molecular Biology and Biotechnology Research Center, Research Institute of Natural Science, Gyeongsang National University, Jinju, Republic of Korea; 2 College of Pharmacy, Division of Life and Pharmaceutical Sciences and National Core Research Center for Cell Signaling and Drug Discovery Research, Ewha Womans University, Seoul, Republic of Korea; University of Akron, United States of America

## Abstract

Human leukotriene A4 hydrolase (hLTA4H), which is the final and rate-limiting enzyme of arachidonic acid pathway, converts the unstable epoxide LTA4 to a proinflammatory lipid mediator LTB4 through its hydrolase function. The LTA4H is a bi-functional enzyme that also exhibits aminopeptidase activity with a preference over arginyl tripeptides. Various mutations including E271Q, R563A, and K565A have completely or partially abolished both the functions of this enzyme. The crystal structures with these mutations have not shown any structural changes to address the loss of functions. Molecular dynamics simulations of LTA4 and tripeptide complex structures with functional mutations were performed to investigate the structural and conformation changes that scripts the observed differences in catalytic functions. The observed protein-ligand hydrogen bonds and distances between the important catalytic components have correlated well with the experimental results. This study also confirms based on the structural observation that E271 is very important for both the functions as it holds the catalytic metal ion at its location for the catalysis and it also acts as N-terminal recognition residue during peptide binding. The comparison of binding modes of substrates revealed the structural changes explaining the importance of R563 and K565 residues and the required alignment of substrate at the active site. The results of this study provide valuable information to be utilized in designing potent hLTA4H inhibitors as anti-inflammatory agents.

## Introduction

**Figure 1:**
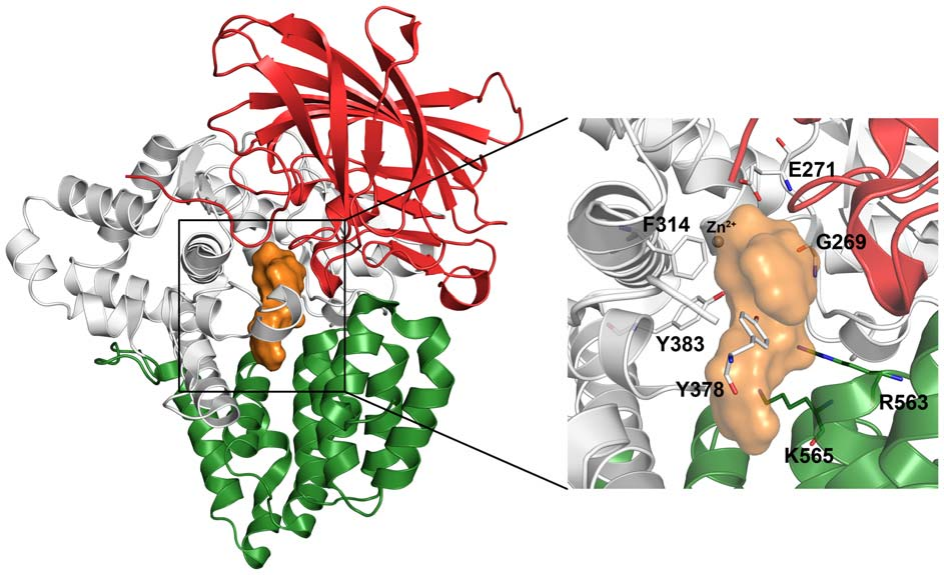
The 3D structure of human LTA4H enzyme. The N-terminal (red), central catalytic (white), and C-terminal (green) domains are shown. The active site located at the center of three domains is occupied with a tripeptide (orange surface). The zoomed view shows the important catalytic residues (sticks) involved in interacting both the substrates. Hydrogen atoms are hidden for a clear view.

Leukotriene cascade is associated with the biosynthesis of variety of leukotrienes (LT) from the phospholipids of the nuclear membrane of the leukocytes [1]. The LTs are a group of lipid mediators associated with acute and chronic inflammatory diseases such as asthma, rhinitis, psoriasis, chronic obstructive pulmonary disease, and atherosclerosis [2]–[13]. Cytosolic phospholipase A2 (cPLA2) hydrolyzes the ester bond present in s*n-2* position of phospholipids and yields lysophospholipids and free fatty acid, arachidonic acid (AA) [1], [14]. This increases the level of free AA available for the synthesis of inflammatory leukotrienes upon the action of more enzymes. The enzyme 5-lipoxygenase (5-LO) assisted by file-lipoxygenase-activating protein (FLAP) converts the AA into the highly unstable allylic epoxide, leukotriene A4 (LTA4) [15]–[21]. This unstable intermediate is converted into two different products LTB4 and LTC4 by the action of two different enzymes LTA4 hydrolase (LTA4H) and LTC4 synthase (LTC4S), respectively [1], [22]–[25]. The LTC4 is subsequently converted to LTD4 and LTE4 substances by the action of different enzymes. All of these LTB4, LTC4, LTD4, and LTE4 are powerful proinflammatory mediators [1], [26]. The LTA4H, which catalyzes the conversion of LTA4 to the chemotactic agent LTB4, was identified as a bi-functional enzyme capable of processing two highly diverse substrates such as LTA4 (a fatty acid) and peptide through its epoxide hydrolase and aminopeptidase activities [27], [28]. This enzyme was first discovered for its epoxide hydrolase activity and later for its aminopeptidase activity based on the presence of consensus Zn binding motif (HEXXH-X_18_-E), which was found in M1 family of Zn containing aminopeptidases [29]–[32]. The natural peptide substrate for this enzyme is still not known but preference is shown over arginyl di- and tripeptide and can selectively be blocked by the mutation of either E296 or Y383 residues [33]–[36]. Upon the determination of LTA4H crystal structures it was revealed that this enzyme is composed of three domains, a fully beta N-terminal domain, a mixed alpha/beta catalytic domain, and a fully alpha-helical C-terminal domain (Figure 1) [37]–[42]. In terms of the hydrolase activity of the enzyme, D375 from a narrow hydrophobic pocket is specifically required as it is involved in the nucleophilic attack targeting C12 atom of LTA4 [43]. In addition, this residue belongs to the peptide K21 (L365–K385) segment identified by Lys-specific peptide mapping of suicide inactivated LTA4H. The carboxylate moiety of LTA4 was observed to form direct electrostatic interactions with the two positively charged conserved R563 and K565 residues present at the entrance of the active center [28], [44]. These interactions are very much essential in aligning LTA4 along with the catalytic elements of the active site. Based on the mutagenic experiments, E271 residue from another conserved GXMEN motif in the family of zinc peptidases was found to be important for both the functions of the enzyme [14] as the mutagenic replacements abrogated both the activities. A crystal structure of LTA4H with E271Q mutation has revealed only minimal conformational changes and did not explain the loss of enzyme function [14]. It was also suggested that the carboxylate of E271 participates in an acid-induced opening of the epoxide moiety of LTA4 and as N-terminal recognition site in terms of peptide substrates [14], [26], [45]. Some mutagenic experiments have also reported the critical role of R563 residue in epoxide hydrolase reaction by positioning the carboxylate tail along the catalytic elements of the active site [28], [34]. In aminopeptidase reaction, both R563 and K565 residues co-operate each other to ensure the necessary binding strength and productive alignment of the substrate. Altogether, R563 plays important roles in both the functions of the enzyme whereas K565 residue assists R563 in catalyzing the peptide substrate but not in hydrolase reaction, which catalyzes the fatty acid substrate [27]. These residues were reported to be the common carboxylate recognition site for both lipid and peptide substrates in the active site of LTA4H [27]. Mutations of R563 to any other amino acid including a conservative replacement of R by K preserving the positive charge abolished the enzyme function but exhibited a significant residual aminopeptidase activity [27]. Crystal structure with R563A mutant could not reveal any structural changes explaining the complete loss of catalytic activity. It was also reported that esterified LTA4 cannot be the substrate of the enzyme and this phenomenon was explained with the steric hindrance [46], which also proved that a free carboxylate group of LTA4 is critical for the hydrolase function. The other carboxylate recognition residue K565 located in a way that it can also involve in carboxylate recognition but its mutagenic replacements have not decreased the epoxide hydrolase activity [27]. The difference in observed aminopeptidase activities between the wild type and K565 mutants have suggested that K565 is a carboxylate binding site for peptide substrates also [27]. The information of a binding pocket for its ligand is very important for drug design, particularly for conducting mutagenesis studies [47]. In the literature, the binding pocket of a protein receptor to a ligand is usually defined by those residues that have at least one heavy atom (i.e., an atom other than hydrogen) with a distance from a heavy atom of the ligand. Such a criterion was originally used to define the binding pocket of ATP in the Cdk5-Nck5a* complex [48] that has later proved quite useful in identifying functional domains and stimulating the relevant truncation experiments [49]. The similar approach has also been used to define the binding pockets of many other receptor-ligand interactions important for drug design [50]–[54].

**Figure 2:**
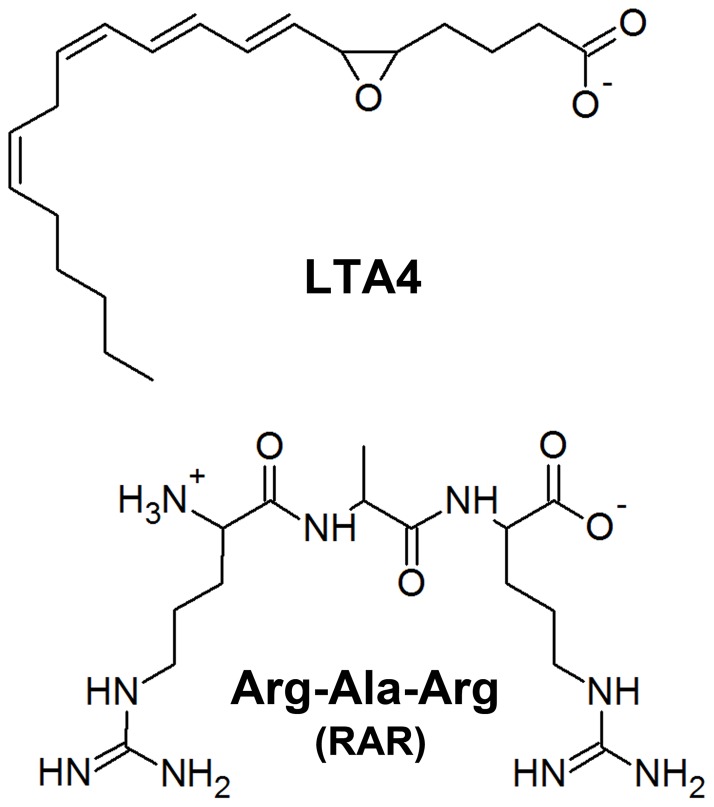
The 2D structures of two diverse substrates of bifunctional enzyme, LTA4H.

This study has focused on structural changes and binding mode differences between wild types and mutant forms of hLTA4H-fatty acid and -tripeptide substrate complexes. Molecular dynamics (MD) simulations of two wild types of the enzyme-substrate complexes and three mutations including E272Q, R563A, and K565A were studied with LTA4 (fatty acid) and Arg-Ala-Arg (RAR, tripeptide) substrate (Figure 2). The binding mode comparison of enzyme-substrate complexes revealed essential structural differences, which were not shown in X-ray structures, explaining the loss of catalytic functions of the enzyme. The overlay of binding modes of LTA4 and RAR substrates has proved the previous assumption of similar but overlapped active site is used in both the functions of the enzyme. The results from this study provide valuable information over the way the two functions of the enzyme are exerted over two substrates of diverse nature. In addition, the structural information obtained from this study can be utilized in structure-based hLTA4H inhibitor design as inhibition of the hydrolase and aminopeptidase functions will lead to the development of anticancer and anti-inflammatory drugs, respectively.

## Materials and Methods

### Preparation of complex structures

As this study was aimed at investigating the binding of fatty acid and peptide substrates of the enzyme, independent hLTA4H-LTA4 (L-LTA4) and hLTA4H-RAR (L-RAR) complexes were prepared. The LTA4 of AA cascade is the natural substrate of the enzyme but no 3D structural information was available for its binding from X-ray studies. Some previous studies have reported the proposed binding of this fatty acid substrate at the active site of the enzyme. In this study, molecular docking methodology using *GOLD* 5.0.1 program was employed to obtain the reliable binding mode of LTA4. The *GOLD* program from Cambridge Crystallographic Data Centre, UK uses a genetic algorithm to dock the small molecules into the protein active site [46], [55]. The *GOLD* allows for a full range of flexibility of the ligands and partial flexibility of the protein. One of the crystal structures of hLTA4H bound with most active inhibitor (PDB ID: 3FUN) solved at high resolution was used in molecular docking. The bound inhibitor of this crystal structure was removed and the apoform of this enzyme was subjected to MD simulations with the parameters discussed below. The representative structure with a RMSD value close to the average structure of last 3 ns of the 5 ns MD simulations was selected and utilized in molecular docking experiments. The active site was defined with a 10 Å radius around the bound inhibitor. The ten top-scoring conformations of every ligand were saved at the end of the calculation. Early termination option was used to skip the genetic optimization calculation when any five conformations of a particular compound were predicted within an RMS deviation value of 1.5 Å. The *GOLD* fitness score is calculated from the contributions of hydrogen bond and van der Waals interactions between the protein and ligand, intramolecular hydrogen bonds and strains of the ligand [56]. Protein-ligand interactions were analyzed using DS and *Molegro Molecule Viewer* [57]. The best pose was selected based on the molecular interactions and the distance between epoxy group of LTA4 and metal ion (Zn^2+^) present in the active site as well as the location of its carboxylate group which interacts with the carboxylate recognition residues R563 and K565. Finally, the enzyme-LTA4 complex was prepared to be used in further steps in this study.

In terms of preparing the enzyme-peptide complex to investigate the aminopeptidase function of LTA4H enzyme, 3D coordinates of the bound tripeptide Arg-Ala-Arg (RAR) in a solved X-ray structure of hLTA4H with a mutation E271Q (PDB ID: 3B7T) was utilized. The *Superimpose Structures* protocol as available in Accelrys Discovery Studio 3.0 (DS) was employed to copy the 3D coordinates of this tripeptide into the representative structure of LTA4H picked from the 5 ns MD simulation by superimposition. This complex was subjected to energy minimization using *Energy Minimization* protocol of DS before considered further in this study.

### Molecular dynamics simulations

Many marvelous biological functions in proteins and DNA and their profound dynamic mechanisms, such as switch between active and inactive states [58], [59], cooperative effects [60], allosteric transition [61], [62], intercalation of drugs into DNA [63], and assembly of microtubules [64], can be revealed by studying their internal motions [65]. Likewise, to really in-depth understand the action mechanism of receptor-ligand binding, we should consider not only the static structures concerned but also the dynamical information obtained by simulating their internal motions or dynamic process. To realize this, the MD simulation is one of the feasible tools. Initial coordinates for the protein atoms were taken from the wild type (WT) and mutant forms of both L-LTA4, L-RAR complex structures. Mutations were introduced at E271Q, R563A, and K565A of the enzyme based on the previous experimental reports to investigate the single active site that catalyzes two different functions upon diverse substrates [14], [45]. The protonation states of all ionizable residues were set to their normal states at pH 7. Eight MD simulations were performed for systems including WT and mutant forms of L-LTA4 and L-RAR complexes (Table 1). All MD simulations were performed with GROMOS96 forcefield using GROMACS 4.5.3 package running on a high performance linux cluster computer [66], [67]. During the MD simulations, all the protein atoms including divalent metal ion (Zn^2+^) were surrounded by a cubic water box of SPC3 water molecules that extended 10 Å from the protein and periodic boundary conditions were applied in all directions. The systems were neutralized with Na^+^ and Cl^−^ counter ions replacing the water molecules and energy minimization was performed using steepest descent algorithm for 10,000 steps. A 100 ps position restrained MD simulations were performed for every system followed by 5 ns production MD simulations with a time step of 2 fs at constant pressure (1 atm), temperature (300 K). The electrostatic interactions were calculated by the PME algorithm and all bonds were constrained using LINCS algorithm. A twin range cutoff was used for long-range interactions including 0.9 nm for van der Waals and 1.4 nm for electrostatic interactions. The snapshots were collected at every 1 ps and stored for further analyses of MD simulations. The system stability and behavior of the catalytic structural components present in every system were analyzed using the tools available with GROMACS 4.0.5 and PyMol programs.

**Table 1:** The details of all systems subjected to MD simulations.

System	Substrate	Mutation	Water molecules	Counter-ions
L-LTA4	LTA4	-	42979	11 Na+
L-LTA4	LTA4	E272Q	42980	10 Na+
L-LTA4	LTA4	R564A	42981	12 Na+
L-LTA4	LTA4	K566A	42979	12 Na+
L-RAR	RAR	-	42993	8 Na+
L-RAR	RAR	E272Q	42994	7 Na+
L-RAR	RAR	R564A	42995	9 Na+
L-RAR	RAR	K566A	42990	9 Na+

## Results and Discussion

### Molecular mechanisms and enzyme-substrate complexes

**Figure 3:**
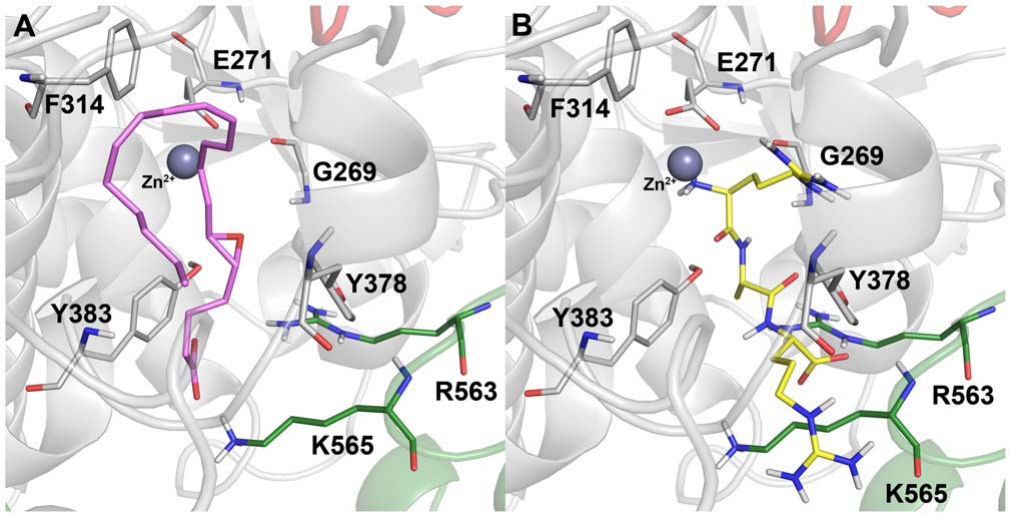
The initial binding poses. (A) LTA4 and (B) RAR selected from the molecular docking studies to be subjected to MD simulations.

Surprisingly, the LTA4H enzyme catalyzes both hydrolase and aminopeptidase functions over fatty acid and peptide substrates utilizing the same active site. In order to obtain deeper insight upon this unique characteristic of the enzyme, a set of MD simulations were performed with WT and mutated enzyme-substrate complex structures. The natural epoxy substrate LTA4 of arachidonic acid pathway, which is converted to LTB4 upon the action of the enzyme, was selected as fatty acid substrate to investigate the hydrolase function of the enzyme. In the other hand, RAR tripeptide that is reported to be the preferred peptide substrate of the enzyme [14] was selected to investigate the aminopeptidase function of the enzyme. The L-LTA4 complex was prepared through the molecular docking methodology whereas L-RAR complex was prepared by copying the 3D coordinates of RAR from the X-ray crystal structure of LTA4 (Figure 3). The representative structure obtained from the MD simulation of LTA4H-apoform was used in preparing both the complex structures to compare the structural changes effectively with no artifacts. From the reported site-directed mutagenesis experiments, three amino acid residues from the catalytic active site of the enzyme were predicted to be very important for the enzymatic activities of the enzyme. These residues include one negatively charged E271 residue from the central catalytic domain and two positively charged R563 and K565 residues from the C-terminal domain. Studies mentioned that mutation of this negatively charged residue to a neutral glutamine (E271Q) has completely abrogated both the catalytic activities of the enzyme. But the crystal structure solved with this mutation (PDB ID: 1H19) could not report any structural or conformational changes causing this drastic change in the catalytic activity [14]. It was also proposed that metal (Zn^2+^) ion present close to the epoxide moiety of LTA4 acts as a weak Lewis acid to activate and open the epoxide ring. It continued to explain that the E271 located in proximity to Zn^2+^ also participates in this acid-induced opening of the epoxide ring of LTA4. It was also reported that E271 acts as the N-terminal recognition point in stabilizing the peptide substrates in terms of aminopeptidase reaction of the enzyme. The positively charged residues R563 and K565 were predicted as the carboxylate recognition sites for both the substrates of the enzyme. All mutations of R563 including R563K, which preserved the positive charge, have resulted in complete loss of catalytic functions of the enzyme. The R563K mutant has shown a significant aminopeptidase activity. All these mutations of R563 leading to tremendous change in enzyme functions did not reveal any structural changes explaining the loss of activities. Especially in epoxide hydrolase reaction, the role of R563 was presumed to position the carboxylate tail of the substrates along the catalytic components of the active site [45]. In terms of aminopeptidase reaction, both the positively charged (R563 and K565) residues help each other in aligning the substrate with the catalytic elements and maintaining the binding strength. The mutations K565A and K565M lacking the positive charge have reduced the aminopeptidase and revealed that this positively charged residue assists R563 in carboxylate recognition in aminopeptidase reaction [27]. Despite of this information over the importance of E271, R563, and R565, the structural changes explaining the catalytic activities of the enzyme are lacking. The results of MD simulations of WT and mutant enzyme-substrate complexes discussed in this study will provide a deeper insight from the structural perspective.

### Overall structural stability of the systems

**Figure 4:**
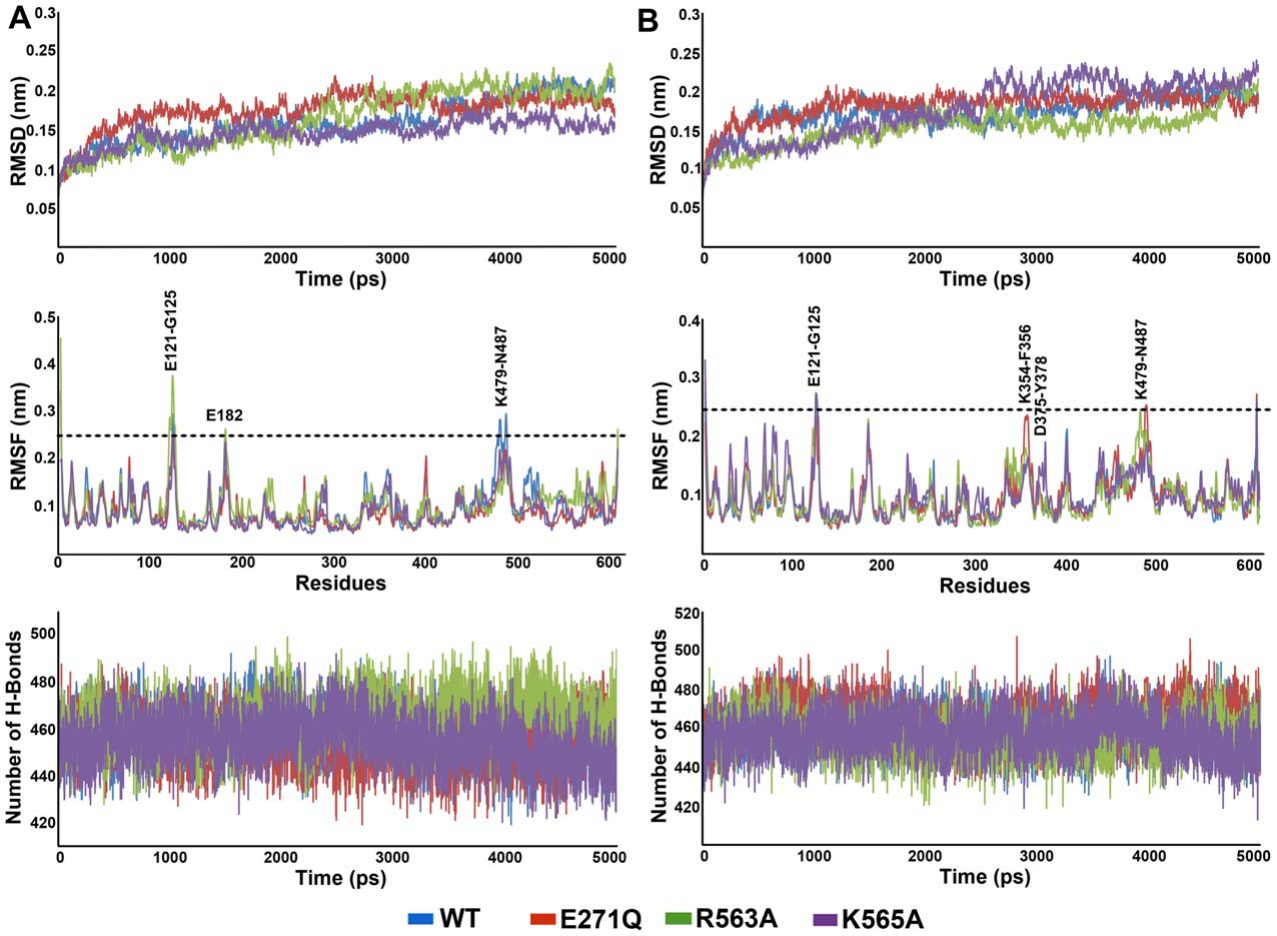
Basic analyses to investigate the overall of behavior of the systems during the MD simulations. The RMSD, RMSF, and number of intramolecular hydrogen bonds are shown for (A) L-LTA4 (B) L-RAR systems.

The overall stability analyses are considered important to note that the systems did not undergo any unusual changes during the time scale of simulation because of erratic system preparation. In this study, root mean square deviation (rmsd), root mean square fluctuation (rmsf), and intra-molecular hydrogen bonds were used in analyzing and comparing the stability of the systems under study (Figure 4).

In terms of L-LTA4 systems, the calculated average rmsd value of K565A system was 0.158 nm which is lower than the rmsd values of WT (0.189 nm), E272Q (0.186 nm), and R563A (0.202 nm) systems (Figure 4A). The R563A system has shown the higher rmsd value and thereby indicating the additional effect of this particular mutation. Despite of these small differences in the rmsd values between the L-LTA4 systems, the systems were stabilized well throughout the timescale of simulation. As another method to investigate the stability of the systems, rmsf values of all systems were calculated during the simulation and plotted. From the plot, it was observed that none of the active site residues were fluctuating higher than 0.2 nm and explained the stable nature of the systems over the time scale of simulation. In addition, the number of intramolecular hydrogen bonds were calculated for all the systems and plotted. The average number of hydrogen bonds revealed that R563A system has formed more number of hydrogen bonds (466.9) compared to WT (452.8), E272Q (451.7) and R565A (450.6) systems which displayed the reduced number of hydrogen bonds. This result also has confirmed the stability of the systems despite of small differences between systems (Figure 4A).

In L-RAR systems, the average rmsd value of K565A (0.212 nm) was higher than other systems, which is completely contrasted to the rmsd value of equivalent L-LTA4 system whereas the R563A system has shown the average lower rmsd value (0.168 nm). The other two systems, WT and E272Q, have shown the same average rmsd value of 0.189 nm from last 3 ns of the simulation time (Figure 4B). In terms of rmsf calculations, the rmsf plot has shown that except D375 and Y378 residues of the active site all other important active site components were stable throughout the simulation. This high fluctuation of these two residues was mainly observed in K565A system. The average number of intramolecular hydrogen bonds during last 3 ns of the simulation was very similar in all the systems. At the end of the simulation time, the R563A and K565A systems started losing their hydrogen bonds and thereby became less intact compared to other systems. All the systems were investigated for the stability and found to be well stabilized during the simulation. Thus the representative structures close to the least rmsd value of each system was obtained and used in structural comparison.

### Distance and hydrogen bond analyses

**Figure 5:**
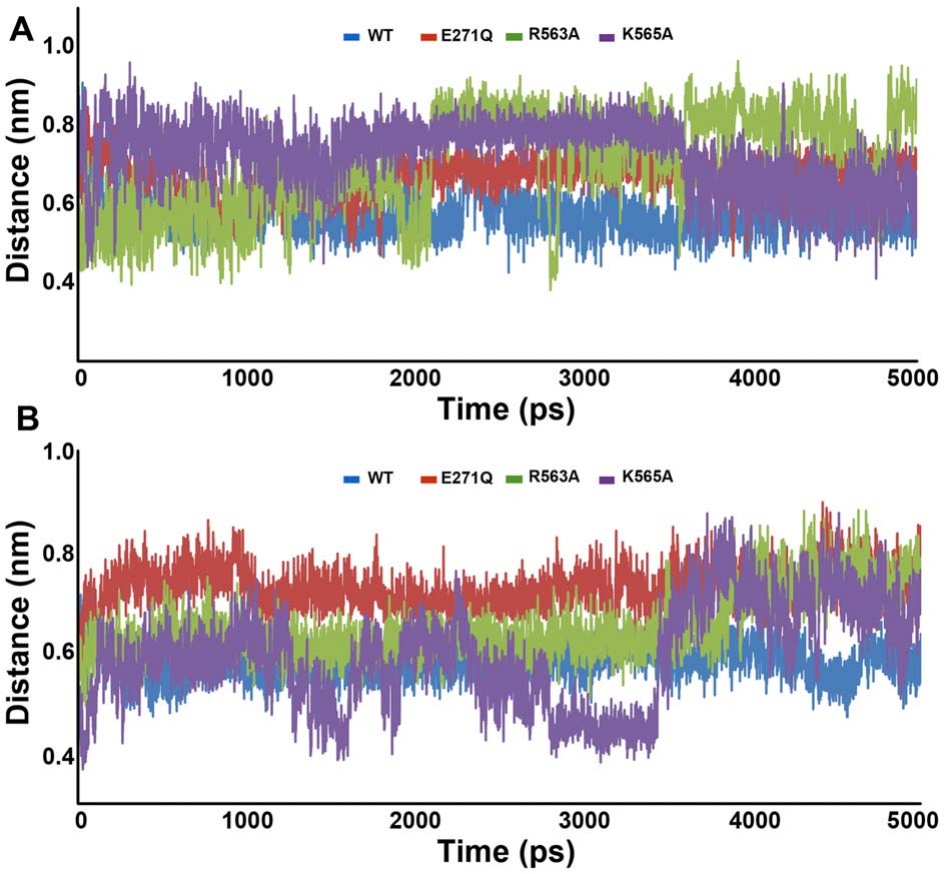
The calculated distances between the catalytically important moieties of the system. (A) Distance between the metal ion and the epoxy oxygen atom of LTA4. (B) Distance between the metal ion and carbonyl oxygen atom of the N-terminal peptide bond of RAR.

The distance between the most important metal (Zn^2+^) ion and oxygen atom of the epoxy ring in case of L-LTA4 systems is very important for the hydrolase function of the enzyme. The average distance value observed in WT system (0.56 nm) was lower compared to the mutant systems. Among mutant systems R563A system has shown the higher average distance value of 0.78 nm whereas E271Q and K565A systems have shown similar average distance value of 0.69 nm and 0.63 nm, respectively. During the end of the simulations, this distance in E271Q and K565A systems has reduced close to the distance of WT but R563A has maintained higher distance until the end (Figure 5A). In terms of L-RAR systems, the distance between the same metal ion and carbonyl oxygen atom of N-terminal peptide bond was measured and compared between the L-RAR WT and mutant systems. As observed in L-LTA4 systems, WT of L-RAR systems has maintained the lower average distance value of 0.59 nm whereas all the mutant systems have shown higher distance values indicating the disturbance of optimal distance for the catalysis. The E271Q and R563A systems have shown the average distance values of 0.74 nm and 0.70 nm, respectively whereas K565A system has displayed an average distance value of 0.64 nm (Figure 5B).

**Figure 6:**
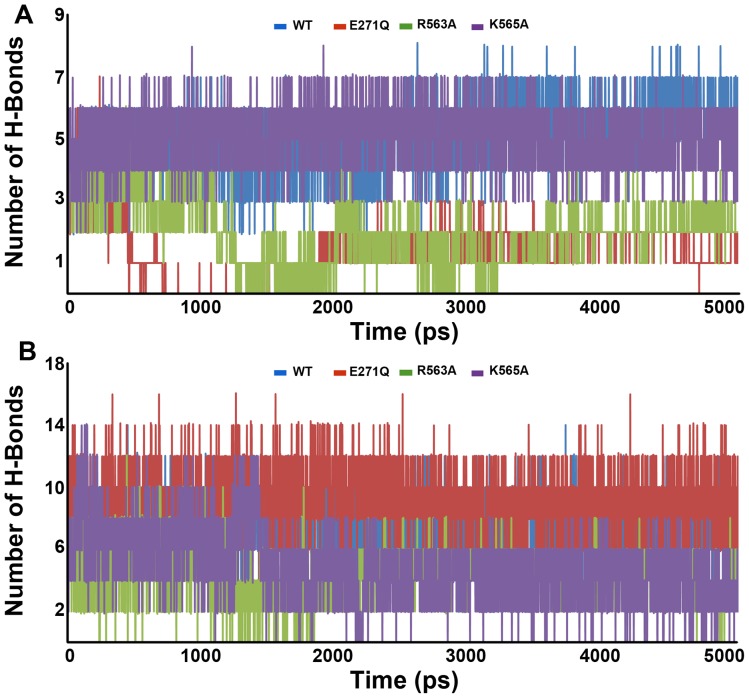
The distribution of number of intermolecular (enzyme-substrate) hydrogen bonds during MD simulation. (A) Number of hydrogen bonds between the protein and LTA4. (B) Number of hydrogen bonds between the protein and RAR.

The hydrogen bonds formed between the protein and bound ligands were also calculated for all the systems under study to investigate the molecular interactions that are lost during the mutations. In L-LTA4 systems, WT system has formed high number of average hydrogen bonds (5.7) compared to the mutant systems. The K565A system has shown an average number of hydrogen bonds of 5.0 whereas E271Q and R563A systems have formed only 1.7 and 1.8 average number of hydrogen bonds with the bound ligand (Figure 6A). This reduced number of hydrogen bonds correlate well with previously reported loss of hydrolase activity of the enzyme in E271Q and R563A mutated systems [14], [45]. In terms of L-RAR systems, the E271Q system has formed more number of average hydrogen bonds (8.7) with the bound RAR. Whereas the WT system has shown an average hydrogen bond value of 8.0, the other mutant systems R563A and K565A have shown the average hydrogen bond values of 4.3 and 3.9, respectively (Figure 6B). This result of number of hydrogen bond values observed between protein and RAR has proved the importance of both R564 and K565 to maintain the substrate alignment in the active site and the binding strength of the substrate. But the high number of observed hydrogen bonds in E271Q system does not correlate with the observed loss of catalytic activity due to the mutation of E271. This indicated that other structural disturbances script the loss of catalytic function of the enzyme.

### Binding mode analyses

#### L-LTA4 systems

**Figure 7:**
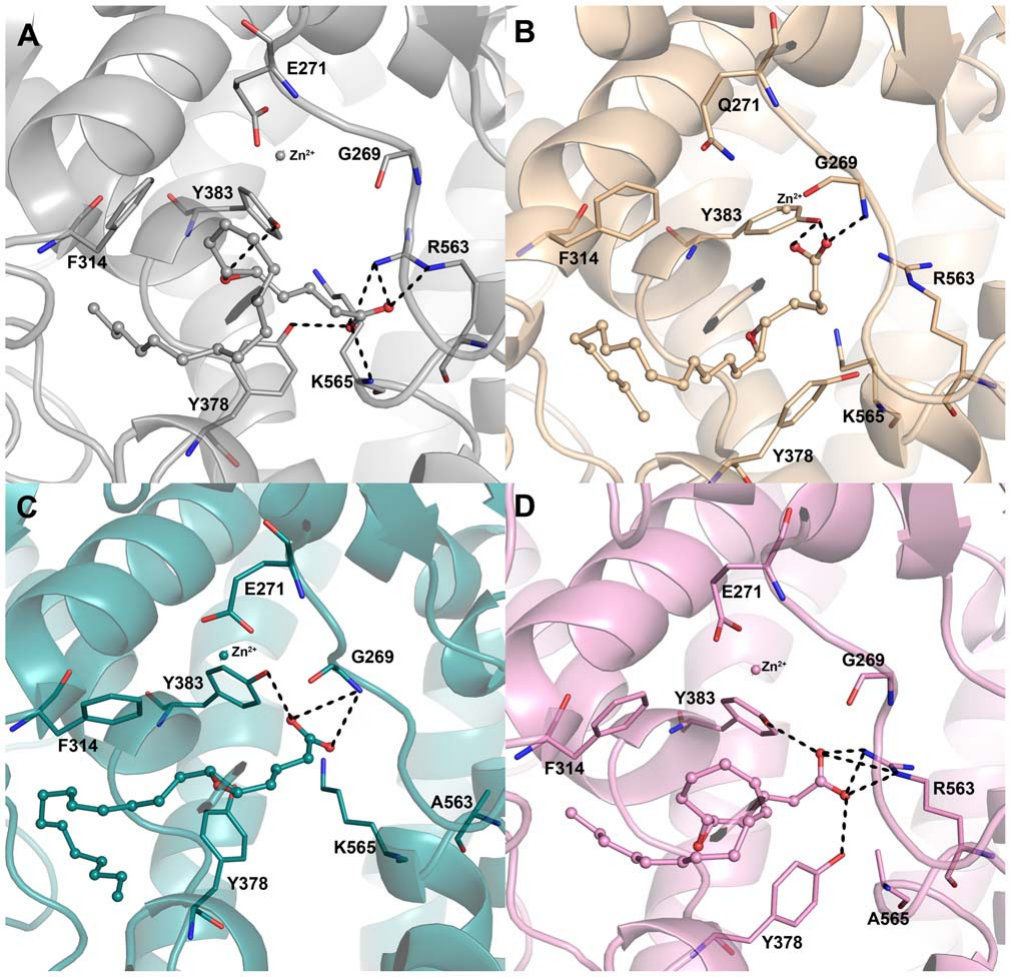
The binding modes of LTA4 at the active site of the enzyme in L-LTA4 systems. The binding mode of LTA4 in (A) WT (grey), (B) E271Q (wheat) (C) R563A (deep teal), and (D) K565A (pink) systems. The amino acid residues and LTA4 are shown in think stick and ball-stick forms, respectively. The metal ion (zinc) present at the active site is shown in sphere form.

In L-LTA4 systems, the binding mode of LTA4 in WT system has formed hydrogen bonds with Y383 through the oxygen atom of epoxy ring and the distance between this oxygen atom and the metal ion was maintained in a closer distance compared to that of mutant systems. The carboxylate group of LTA4 in WT system was well recognized by both the positively charged residues R563 and K565 which are reported to be the carboxylate recognition sites. Because of this recognition the carboxylate group has formed strong hydrogen bond interactions with R563 and K565. The distance between the carboxylate of E271 and metal (Zn^2+^) ion was maintained in close vicinity for the reported acid-induced catalytic reaction. The other residue Y378 has also formed a hydrogen bond interaction with the carboxylate group of LTA4 (Figure 7A). In E271Q system, the binding mode of LTA4 is so different from that of WT system. Because of the uncharged nature of the mutation (E271Q) the metal ion has slightly moved towards the carboxylate group of LTA4, which has also mutually moved towards the metal ion. This movement of carboxylate group of LTA4 has moved the central epoxy group of LTA4 further down and made it inaccessible by the catalytic metal ion (Figure 7B). This change in the E271 and distance between Zn^2+^ and epoxy group (0.69 nm) can be directly correlated with the loss of activity (Figure 5A). The hydrogen bonds formed with R563 and K565 residues were completely lost because of this mutation. In R563A system, though E271 residue has maintained its hold on the metal ion because of the absence of R563, the important carboxylate recognition site, the LTA4 has moved backwards into the hydrophobic cavity formed by hydrophobic residues such as W311, F362, K364, L365, V366, V367, and V381 (Figure 7C). This change has not only brought the distance between Zn^2+^ and the epoxy ring of LTA4 higher (0.78 nm) compared to any other L-LTA4 systems but also made hydrogen bonding with K565 impossible. In terms of K565A system, the binding mode of LTA4 was similar to that of WT system. Regardless of mutated K565 the hydrogen bonds were maintained with R563 but still the distance between Zn^2+^ and epoxy ring of LTA4 was higher (0.68 nm) in this mutant system as well. The hydrogen bond between LTA4 and Y378 was also maintained as observed in WT system (Figure 7D). This observation also correlated the experimental observation that K565A mutation reduces the activity but does not abolish it.

#### L-RAR systems

**Figure 8:**
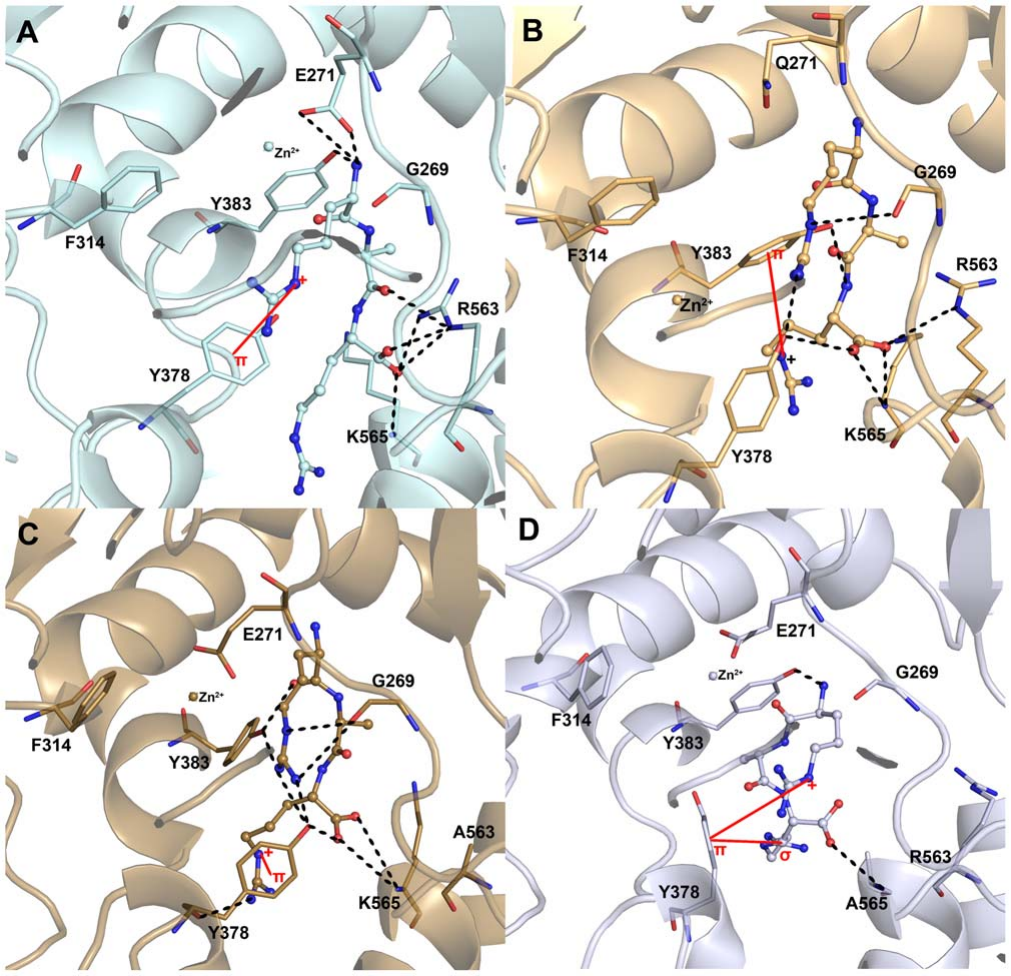
The binding modes of RAR at the active site of the enzyme in L-RAR systems. The binding mode of RAR in (A) WT (cyan), (B) E271Q (light orange) (C) R563A (sand), and (D) K565A (blue white) systems. The amino acid residues and RAR are shown in think stick and ball-stick forms, respectively. The metal ion (zinc) present at the active site is shown in sphere form.

The binding modes of the tripeptide RAR in all systems were observed and compared to investigate the changes due to the mutated residues. More number of hydrogen bonds was observed between the protein and bound substrate compared to the L-LTA4 systems because of the high number of polar hydrogen in the peptide substrate. In the WT system, strong molecular interactions were observed through the hydrogen bonds and π-cation interactions formed between protein and substrate (Figure 8A). The C-terminal carboxylate which is equivalent to the carboxylate of LTA4 has formed strong hydrogen bond interactions with both positively charged residues R563 and K565, the carboxylate recognition sites. These interactions mainly hold the RAR at the active site and improve its binding strength. As reported, the N-terminal amino group interacted with the E271 which is the N-terminal recognition site for the peptide substrates. These interactions altogether brings the carbonyl oxygen atom of N-terminal peptide bond close to the catalytic metal ion. A π-cation interaction was formed between the side chain of C-terminal Arg residue and Y378, which was found highly fluctuating in rmsf analysis. This π-cation interaction between the same atoms was observed in R563A and K565A systems as well whereas it was between Y383 and C-terminal Arg residue in E271Q system (Figure 8). The binding mode of the substrate in E271Q system was different to that of WT system. The strength of the hydrogen bonds with R563 and K565 has become weak in this system because of the conformational changes of both carboxylate of substrate and R565 residue (Figure 8B). The metal ion located in the active center was thrown away from its initial position because of the absence of negatively charged E271. This behavior observed in this system clearly reveals that E271 acts as a hook to hold the Zn^2+^ ion in the active site. In terms of R563A system, the observed binding mode of this system is similar to that of E271Q system (Figure 8C). The metal ion was hooked by the presence of E271 residue but still the distance between Zn^2+^ and the carbonyl oxygen atom of N-terminal peptide bond was high compared to that of WT. The hydrogen bonds were formed between Y378, Y383, and G269 residues. Surprisingly, two hydrogen bonds were formed with K565 residue in absence of R563. This is different compared to that of the equivalent L-LTA4 system where the hydrogen bonds with both the positively charge residues were completely lost. This observation indicates the importance of K565 in assisting R563 in carboxylate recognition during peptide binding. In K565A system, the binding mode of the substrate is folded and completely different to the other systems. The hydrogen bonds were observed only with Y383 and A565 residues along with an additional π-σ interaction between Y378 and C-terminal Arg residue (Figure 8D). No hydrogen bonds were formed with R563 residue, which is one of the positively charged carboxylate recognition site residues. The molecular interactions observed in R563A and K565A systems revealed that both the residues are important for recognizing the carboxylate group and aligning the peptide substrate along the catalytic elements as reported.

### Active site structural changes

#### L-LTA4 system

**Figure 9:**
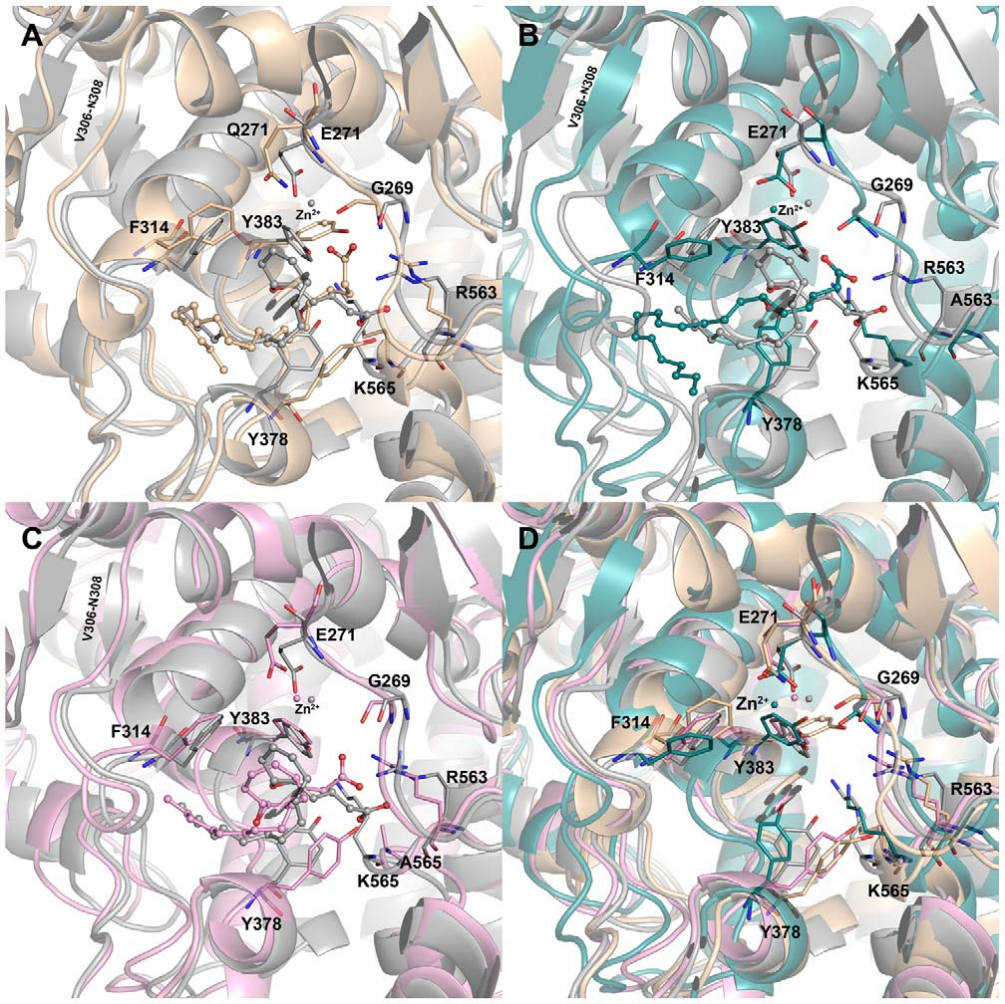
Overlay of WT and mutant systems of L-LTA4 complexes to observe the structural changes. (A) WT and E271Q systems, (B) WT and R563A systems, (C) WT and K565A systems, and (D) all L-LTA4 systems. The WT, E21Q, R563A, and K565A systems are shown in grey, wheat, deep teal, and pink colors, respectively. The amino acid residues and LTA4 are shown in think stick and ball-stick forms, respectively. The metal ion (zinc) present at the active site is shown in sphere form.

The overlay of each mutant system with the WT system has allowed observing the structural changes occurred because of the mutations (Figure 9). In E271Q system, the loop of G269 was fluctuating and moved into the active site when compared to other systems. This change observed in this loop could be because of the newly formed hydrogen bond between G269 and the tilted carboxylate group of LTA4, which was found to be a response to the metal ion that lost the interaction with mutated E271. The carboxylate group of LTA4 and terminal NH_2_ of R563 moved away from each other (2.7 to 8.4 Å) because of the missing E271. A short beta sheet formed by the residues V306-N308 of HEXXH-(X)_18_-E motif that possess two catalytically conserved histidine residues coordinating with Zn^2+^ ion disappeared in E271Q system. Another helix followed by this short beta sheet was extended by four amino acids W311-F314 making the important F314 slightly backward from the active center (Figure 9A). Two tyrosine residues Y378 and Y383 located opposite to each other in the active site were highly fluctuating in this system to adjust the binding of the LTA4. The K565 residue present in the loop has become a part of the long helix originally formed by T567-A575 residues during the simulation of E271Q system. This change slightly drew back the K565 residue from the active site. The other important positively charged residue did not show any structural changes during the simulation. The R563A system has shown different structural changes compared to E271Q system. The loop containing G269 has shown slight fluctuation only at the location of G269 because of the hydrogen bonds formed between the carboxylate of LTA4 and G269 but the lower part of the loop was stable unlike E271Q system (Figure 9B). The short helix formed by V306-N308 residues was maintained in this system and the helix containing F314 was extended but kept for the same length during the simulation. The region (W311-F314) that turned an extended helix was highly fluctuating in this system because of the moving alkyl part of LTA4. This backward movement buried the alkyl part into the hydrophobic pocket, formed by a mixture of aliphatic and aromatic hydrophobic residues (W311, F362, K364, L365, V366, V367, and V381), was observed because of the missing interactions with R563 residue (not shown in figure). Unlike E271Q system, Y378 residue has shown only slight side chain movement as a response to the moving LTA4 whereas Y383 did not show any fluctuations from its initial position. The same helix extension was observed as in E271Q system and thus K565 was included in helix formed by T567-A575 residues. The missing R563 led to the loss of correct alignment of LTA4 along the catalytic elements and severe instability of the binding mode of LTA4. In the final mutant (K565A) system, the G269 loop was completely stable and no hydrogen bond interaction was observed between LTA4 and G269 residue. The short helix of V306-N308 disappeared during the simulation of K565A system as observed in E271Q system (Figure 9C). The helix of F314 was extended as seen in R563A system and thus has shown the mixed characteristics of E271Q and R563A systems. The Y378, one of the oppositely located pair of tyrosine residues, has fluctuated highly in this system. The binding mode of LTA4 was quite similar to that of WT except its carboxylate group, which moved back because of the missing hydrogen bonds from K565 residue. But the hydrogen bonds with R563 were maintained and thus kept the alignment of LTA4 along with the catalytic elements. The overlay of active sites of all the systems have made clear about the structural changes where Y378 was observed to be fluctuating differently in each system maintaining a close distance with the substrate (Figure 9D). Thus Y378 residue, along with the carboxylate recognition site residues R563 and K565, can play a key role in aligning the substrate at the active site.

#### L-RAR systems

**Figure 10:**
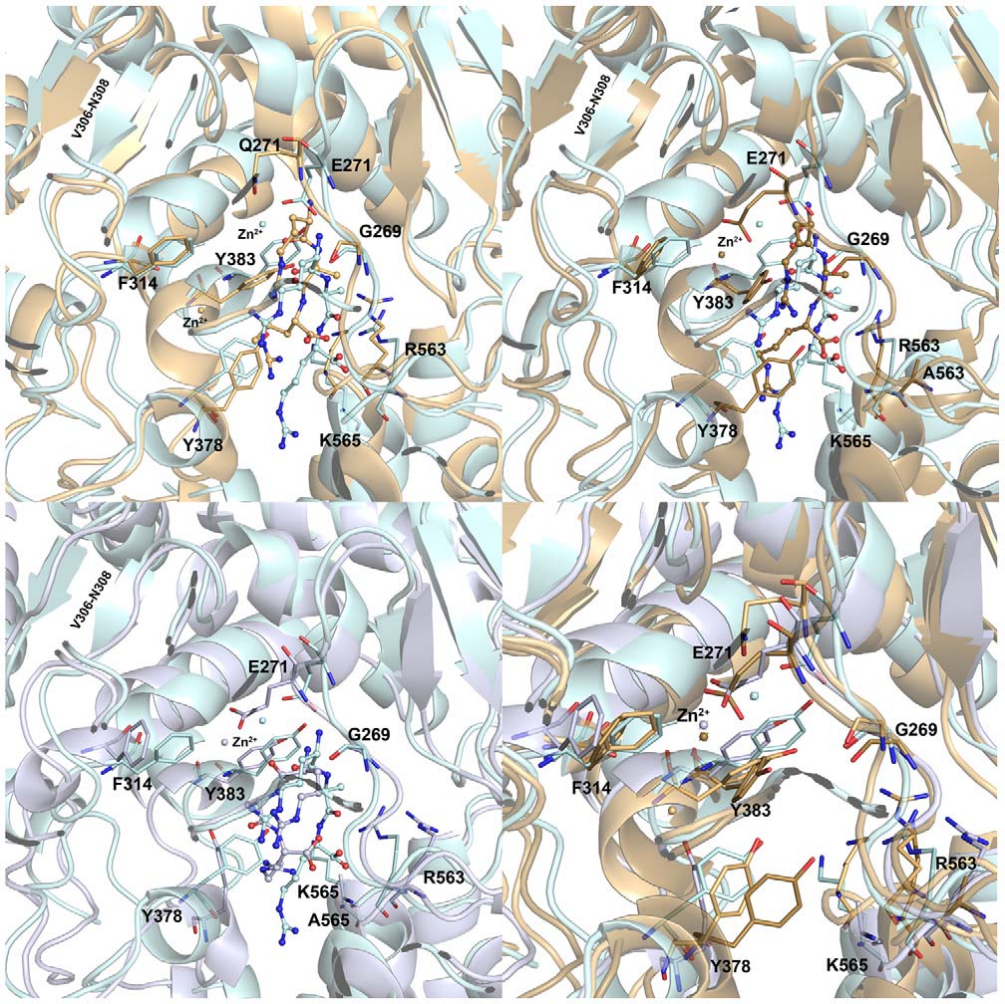
Overlay of WT and mutant systems of L-RAR complexes to observe the structural changes. (A) WT and E271Q systems, (B) WT and R563A systems, (C) WT and K565A systems, and (D) all L-RAR systems. The WT, E21Q, R563A, and K565A systems are shown in grey, wheat, deep teal, and pink colors, respectively. The amino acid residues and RAR are shown in think stick and ball-stick forms, respectively. The metal ion (zinc) present at the active site is shown in sphere form.

Comparison of active site residues of WT and E271Q systems using the representative structures obtained from the simulations revealed the structural changes led to the differences in the catalytic activity of the enzyme (Figure 10). The overlaid WT and E271Q structures have shown the difference in the locations of catalytically important Zn^2+^ ion in the catalytic center (Figure 10A). The uncharged nature of the mutant residue Q271 the metal ion has lost the important interaction and moved far away from its original location. The distance between the Zn^2+^ ion and the oxygen atom of the N-terminal peptide bond was so high compared to the WT system. Thus the catalytic aminopeptidase reaction becomes impossible in E271Q system. The helix containing Y383 was extended during the simulation E271Q system moving Y383 backward from the active site. This movement of Y383 has formed π-cation and a hydrogen bond interactions with the C-terminal part of RAR whereas in WT system this residue has formed a hydrogen bond interaction with the N-terminal amino group. The other tyrosine residue Y378, which was found to be guiding the substrate along with the carboxylate recognition site residues, has formed hydrogen bond with the carboxylate of RAR. The binding mode of RAR observed in E271Q system has shown only weak hydrogen bonding interactions with R563 as it was moving away from it. The helix formed by K565-A575 residues was shortened slightly leaving K565, one of the carboxylate recognition residues, as a part of loop making it more flexible. But this residue has maintained hydrogen bond interactions with the peptide substrate through its carboxylate group. This change observed in helix containing K565 is different from that of L-LTA4 systems. The K565 is the key residue that assists the other positively charged residue R563 to maintain the proper alignment of RAR in the active site whereas in LTA4 binding K565 is not required to assist R563 residue [27]. Moreover, the absence of negatively charged E271 residue also played a major role in observed loss of catalytic activity. The R563A mutation has caused a high fluctuation of E271 that moved far from the N-terminal amino group of RAR and makes it impossible to act as N-terminal recognition site (Figure 10B). The interacting distance between Zn^2+^ and E271 was maintained in this mutation. The helix extension was observed near Y383 as displayed in E271Q system and this changed the flexibility of Y383 in the active site. The other tyrosine residue Y378 was highly fluctuating in this mutant system compared to any other systems and formed strong π-cation interaction than it is in WT system. The C-terminal part of RAR substrate has slightly went back as it missed the strong interactions from R563 but the alignment was almost maintained as observed in WT system except the side C-terminal side chain of RAR. Interestingly, K565 has taken the location of R563 in this mutant system to maintain the hydrogen bonds with the carboxylate of RAR and there was no change observed in the K565-A575 helix as observed in E271Q system. The K565A system also has shown some structural changes that were not observed in other L-RAR systems (Figure 10C). The short helix (V306-N308) that has shown structural changes in L-LTA4 system disappeared in K565A system and the F314 helix was extended including W311-F314 residues. These changes were observed in other L-RAR systems. The extension of helix has drawn F314 residue back from the active site center. A folded binding mode of RAR was observed in K565 system much different from that of WT and other mutant L-RAR systems. Though R563 is present, the carboxylate group of RAR has moved back from its original position and formed π-interactions with Y378 and completely lost interactions with R563. This observation completely correlates with the observed activity and the reported statement that K565 assists R563 to act as carboxylate recognition site in aligning the substrate along the catalytic elements of the enzyme. The overlay of all L-RAR systems revealed that along with E271, R563, K565 residues, Y378 and Y383 were also important in keeping the peptide substrate aligned within the active site (Figure 10D). As observed in L-LTA4 systems, Y378 residue has acted as a baffle to control the binding modes of the substrates.

This part of the study has documented various structural changes explaining the differences in activities between WT and mutated forms of the enzyme bound to its two different substrates which were not determined by the X-ray crystallography so far (Table S1).

### Binding modes of LTA4 and RAR substrates

**Figure 11:**
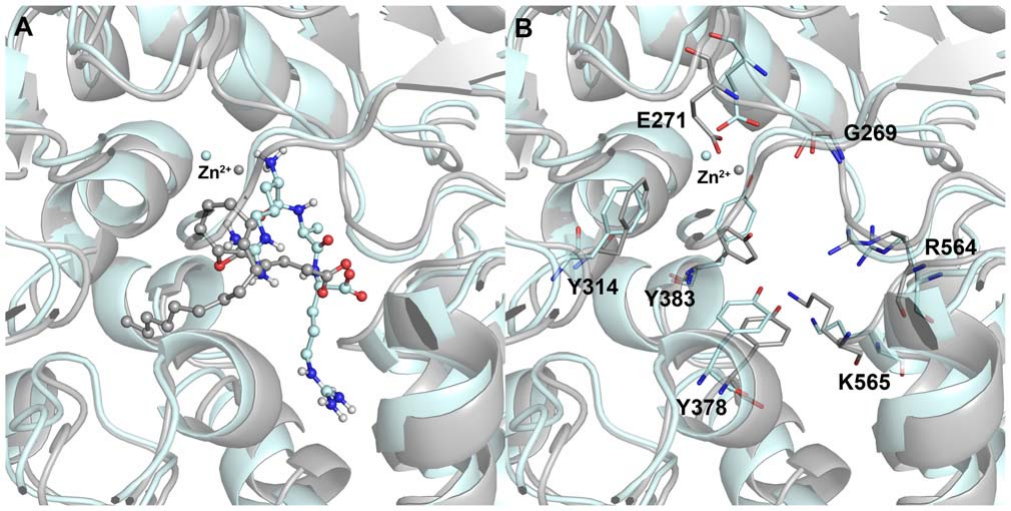
The binding modes of two substrates in the active site of the WT enzyme. (A) LTA4 and RAR substrates shown in grey and cyan at the active site (B) the catalytic active site residues are shown in thin stick form.

The binding modes of fatty acid and peptide substrates that are catalyzed by hydrolase and aminopeptidase functions of the enzyme using same active site were compared. The overlay of two WT systems has given the overview of which parts of the active site were occupied by these two highly diverse substrates. Both the substrates bind perpendicular to each other occupying majorly the different portion of the active site and sharing the carboxylate recognition sites in common (Figure 11A). The long alkyl part of LTA4 was snuggly bound into the hydrophobic pocket formed by a mixture of aliphatic and aromatic residues including W311, F314, K364, L365, V367, Y378, and V381. The side chain of C-terminal Arg residue of RAR was fit into the small cavity formed by F356, Y378, S379, M564, K565, and R568 residues. The overlay of active site residues has shown very few structural changes between the WT systems of L-LTA4 and L-RAR systems including the side chain movements of Y378, Y383, and K565 residues (Figure 11B). The K565 residue was present in the loop in WT of L-LTA4 system and in the extended helix in WT of L-RAR system and thereby changing the flexibility and interacting behavior of K565. This adds explanation to the importance of K565 residue in assisting R563 residue in aligning the peptide substrate whereas this residue is not required in aligning fatty acid substrate.

### Conclusion

In this study MD simulation methodology was used to simulate hLTA4H enzyme complexed with its two diverse substrates along with mutated key residues. The aim of this study was to investigate the structural and conformational changes in the bi-functional active site of the enzyme reflecting on the catalytic activity. This was considered very important and necessary to script the reasons for the observed loss of activity due to particular mutations as the solved X-ray structures failed to show the structural changes. Eight systems including two WT, enzyme-LTA4 and enzyme-RAR complexes along with three independent mutations (E271Q, R563A, and K565A) in each complex were simulated in this study. The observed hydrogen bond interaction network and distance between the catalytically important atoms have correlated well with the experimental results. The E271 residue which is considered very important for both functions of the enzyme and E271Q systems have revealed from our study that this residue acts as a hook to hold the catalytic metal ion at its location and also plays a role of N-terminal recognition point for the aminopeptidase function. Both the E271Q systems have lost the expected binding mode of the substrates for the successful catalysis. The other mutant R563A and K565A systems have also revealed the structural changes and binding mode differences explaining the loss of activity in mutant systems. In L-LTA4 systems, the substrate binding mode in R563A system has changed completely that the long alkyl chain of LTA4 was completely buried into the hydrophobic pocket. This difference in binding mode of LTA4 was completely because of the loss of hydrogen bond interaction with R563 residue. In terms of L-RAR systems, the same mutation R563A has affected the binding mode of RAR and N-terminal recognition through E271 residue in peptide binding. Because of this missing N-terminal recognition the catalytic distance between the metal ion and the carbonyl group of the N-terminal peptide bond was high in this system. The K565A systems in both the substrate complexes have shown different structural changes. In L-LTA4 system the binding mode of the substrate was very much similar to the WT explaining the less importance of K565 in LTA4 binding whereas in L-RAR system the binding mode has lost both the N-terminal and C-terminal recognitions leading to the loss of activity. These results obtained from this study can be effectively used in designing future hLTA4H inhibitors as anti-inflammatory and anti-cancer therapeutics.

## Supporting Information

Table S1 **The structural changes which were not seen in experimental studies observed through the MD simulation studies.**
(DOCX)
