# Supplementary material for: Structural Origins for the Loss of Catalytic Activities of Bifunctional Human LTA4H Revealed through Molecular Dynamics Simulations
Source: PLoS One. 2012 Jul 25;7(7):e41063. doi: 10.1371/journal.pone.0041063 (PMC3405069; doi:10.1371/journal.pone.0041063)
Supplement: Table S1 — The structural changes which were not seen in experimental studies observed through the MD simulation studies. (DOCX) [file pone.0041063.s001.docx]

**Table S1.** The structural changes which were not seen in experimental studies observed through the MD simulation studies.

| **System description** | **Identified information through MD simulations** |
| --- | --- |
| L-LTA4 E271Q | Metal (Zn) ion and COO^-^ of LTA4 mutually moved towards each other. |
|  | Initial distance between COO^-^ of LTA4 and R563 which is 2.7 Å has become 8.4 Å. |
| L-LTA4 R563A | Interactions with K565 were lost because of missing R563. |
|  | Alkyl part of LTA4 moved back into the hydrophobic pocket. |
|  | Binding mode of LTA4 is misaligned. |
| L-LTA4 K565A | Similar binding mode to the WT. |
|  | Indicated the less importance of K565. |
| L-RAR E271Q | Fewer interactions compared to WT were observed especially with R563 and K565. |
|  | Lost the metal (Zn) ion at its position. It moved away and interacted with E318 residue. |
|  | Folded binding mode which is very much different from WT was seen. |
| L-RAR R563A | Interactions with E271 were lost but K565 interacts.  Misaligned binding mode was observed. |
| L-RAR K565A | Interactions with E271 were lost. |
|  | R563 could not interact with the protein because of missing K565. This indicated the importance of K565 (unlike LTA4 binding where K565 was not important). |
